# Building trust before the next crisis: lessons from the avian influenza front lines

**DOI:** 10.3389/fpubh.2025.1735139

**Published:** 2026-01-16

**Authors:** Eri Togami, Elizabeth Ashby Guo, Julie Liao, Stephen Ostroff, Jonathan M. Sleeman, Elizabeth L. Mumford

**Affiliations:** 1 Johns Hopkins Bloomberg School of Public Health, Baltimore, MA, United States; 2 National Academies of Sciences, Engineering, and Medicine, Washington, DC, United States; 3 Advisors of Global Health, Springfield, VA, United States; 4 Department of Epidemiology, School of Public Health, University of Pittsburgh, Pittsburgh, PA, United States; 5 Food and Agriculture Organization of the United Nations, Bangkok, Thailand; 6 Faculty of Veterinary Science, Mahidol University, Salaya, Thailand; 7 School of Veterinary Medicine, Department of Comparative Biomedical Sciences, University of Surrey, Guildford, United Kingdom

**Keywords:** avian influenza, H5N1 (avian influenza), incentives, One Health, outbreak response, risk communication, trust, zoonoses

## Abstract

The United States H5N1 outbreak which began in 2022 had widespread human health, animal health, and economic impacts. This outbreak led to the death or depopulation of over 175 million domestic birds and marked the first time that a highly pathogenic avian influenza (HPAI) virus was detected in cattle. Response to this emergency required coordination among various stakeholders in public health and agriculture sectors at federal, state, and local levels. Despite national and local efforts, a lack of trust between stakeholders diminished the efficiency of the response. The National Academies of Sciences, Engineering, and Medicine hosted a webinar featuring four experts in different agriculture sectors to gain their perspectives from the field on building trust during the H5N1 response. Their discussion highlighted the importance of proactive trust-building, communication and transparency, and incentives as catalysts to building trust in advance of a public health crisis. These insights are key for improving responses to future HPAI outbreaks and other health emergencies.

## Introduction

Since 2022, the United States is experiencing an outbreak of highly pathogenic avian influenza (HPAI) due to influenza A H5N1 virus clade 2.3.4.4b (1). This outbreak has affected numerous wild and domestic animal species, resulting in the death or depopulation of over 175 million domestic birds nationally and the first reported illnesses in dairy cows. By September 2025, nearly 1,100 infected dairy herds in 17 states were reported along with 70 human cases, mostly in agricultural workers (2, 3). Though the Centers for Disease Control and Prevention (CDC) ended its emergency response in July 2025 due to a decrease in new cases, H5N1 had not ceased to expand. Certain regions of the United States saw increased detections in poultry flocks after the emergency response ended, and H5N1 was first detected in Nebraska dairy cattle in September 2025 (4). The H5N1 response remains a highly relevant issue in public health as it illustrates the pressing and persistent challenge of building trust among diverse stakeholders in advance of an emergency.

Discussions of research priorities based on heightened concerns of an H5N1 human pandemic highlighted that a lack of trust in government information and policies among impacted stakeholders may decrease uptake of outbreak mitigation measures (5). Effective control of zoonotic diseases such as HPAI requires a One Health approach, entailing close collaboration between human, animal, and environmental health expertise (6). In this outbreak, major stakeholders include government, agricultural, and human health agencies at federal, state, and local levels; owners, managers and workers in commercial and small-scale poultry and dairy farms; dairy, meat, and egg processors; state agricultural extension services providing production expertise to farmers; and veterinarians providing care to sick or at risk animals (7). Lack of trust among these stakeholders can hinder outbreak detection and response and facilitate further spread of the virus.

We define trust as “the willingness of a party to be vulnerable to the actions of another party based on the expectation that the other will perform a particular action important to the trustor, irrespective of the ability to monitor or control that other party” (8). Trust leads a trustor to be willing to take risks in the trustor-trustee relationship, and trust is context specific (8, 9). For example, a farmer may take a risk by reporting a first case of avian influenza in cattle on their farm to state animal health authorities rather than not reporting it. The farmer risks restrictions on production, reputational burden, amid uncertainty of remediation. In another example, state public health authorities may take a risk by communicating uncertainty to the public that they do not yet know the full extent of the outbreak in their state. They risk raising concerns about health consequences to humans including among consumers of animal products, or being perceived as unknowledgeable. The role of trust in farmers’ disease reporting has been studied in Australia, which found that higher levels of trust were associated with higher intentions to report emerging animal diseases to government authorities (10). Given that trust is a context-specific construct, there is a need to explore how trust may play a role in the H5N1 outbreak in North America.

To explore stakeholder perspectives and opportunities to improve trust-building in the current H5N1 response, the National Academies’ One Health Action Collaborative gathered written comments from impacted parties, including public health professionals, livestock producers, and veterinarians. Written comments were solicited online through the National Academies website in early March 2025. The National Academies then hosted a moderated discussion between the chief agricultural officer of a multistate dairy farm and milk processing corporation, a senior official from United Egg Producers, an agricultural safety and education coordinator working with farm workers, and a state veterinarian. The discussion was a two-hour webinar in late March 2025, which was open to the public. The written comments informed the questions that were asked to the panelists. During the moderated discussion, two investigators documented key points discussed by the panelists, as well as questions from the audience as meeting notes. Subsequently, the authors used the information from the written comments and the meeting notes to iteratively synthesize salient themes over the next three months until consensus was reached. This manuscript outlines the key themes of the discussion in which they shared observations of the H5N1 response. The discussants also identified several interlinked considerations and actions to improve responses to future HPAI outbreaks and other health emergencies.

## Proactive trust-building is a strategic imperative

A critical lesson from this outbreak is that trust is difficult to establish during a crisis: It should be built well in advance. As an example, robust public-private-academic partnerships established before a 2014–2015 HPAI outbreak led to improvements in biosecurity practices on poultry farms that are credited with decreasing lateral spread of the virus (i.e., between farms) from 70% during the 2014–2015 outbreak to <20% in the current outbreak (11, 12). The unique spillover to dairy cattle in the current outbreak highlighted the need to engage and promote trust more broadly in the agricultural sector by sharing knowledge across industries, including longstanding practices on poultry farms and experiential insights from affected dairy producers. There are critical operational nuances that differ among agricultural industries, such as interstate cattle movement for rearing or milking compared with single-site rearing in the poultry industry. Proactively building rapport and shared understanding between agricultural stakeholders before an outbreak, when emotional and financial stress are high, can identify and address concerns and allow better design, uptake of, and trust in outbreak control measures. These dialogue would allow poultry producers to understand why depopulation may not be an appropriate or necessary control measure on dairy farms while it is the standard for poultry. Dairy farmers could learn what measures are practical and translatable from poultry to their operations. Such discussions would ideally involve the swine industry, where producers may resist control measures since only a single H5N1 2.3.4.4b cluster has been reported thus far and experiments suggest it may cause only mild disease with limited transmissibility (13–15).

In the current outbreak, it became clear that each agricultural commodity (e.g., poultry layers and broilers, dairy cattle) has different operational intricacies, risk profiles, and information networks. Policy leadership is essential to foster inter-stakeholder collaboration and encourage information sharing not only among government agencies, but also among agricultural co-operative and extension units, commodity groups, non-profit organizations, farmers, and community leaders. Equally important is establishing platforms for affected and unaffected producers, who may not typically work together, to communicate with and learn from each other. Policymakers must invest in understanding and bridging these differences in preparedness planning to promote proactive trust-building among stakeholders—using effective communications, transparency in engagement, and supportive incentives—to yield more efficient responses that reduce the cost and impact of future crises.

## Communication is central to trust-building

Timely, clear, and consistent communication is central to trust-building (16, 17), particularly in times of change such as during health crises or when introducing new policies, an insight reinforced throughout the COVID-19 pandemic. Reliable early messaging, framed clearly to convey seriousness without invoking panic, is especially important to counter misinformation. Providing precise, actionable steps and the rationale behind them, such as the need for personal protective equipment, is key in reinforcing trust and enhancing compliance. Also, honesty regarding uncertainty is critical to sustaining trust as messages may evolve to reflect updated data or analyses.

Different messengers and formats are effective for different target audiences. Trusted individuals, such as veterinarians, outreach workers, bilingual educators, community-based organizations, and agricultural extension units can be reliable partners for successful communication, especially for new disease control measures or policies. They are generally already embedded within their stakeholder communities and are likely met with less skepticism than “outsiders” such as public health agencies whose involvement is limited to a specific outbreak. Furthermore, there is currently a critical gap in maintaining communication among different agriculture sector industries. Some, but not all, dairy producers were able to learn from producers in other states and from avian influenza biosecurity measures in poultry production. Conversely, a lack of dialogue and common ground in response measures can lead to lack of trust among stakeholders, for example, if the burden of biosecurity measures appears to be borne unequally without a full understanding of distinct operational challenges that may be unique to one commodity.

Understanding how different stakeholders function and recognizing the experiences, perceptions of risk and resources of each that affect their response is vital for creating practical, equitable, and effective communications and policies. Producers and workers should be consulted to understand commodity-specific challenges and rationale for industry practices, such as interstate cattle transport, rapidly moving eggs through the commercial chain, and needs of agricultural workers. Otherwise, messaging may be inappropriate for its audience, leading to nonadherence or unintended consequences.

## Transparency catalyzes trust

Alongside effective communication, transparency is a key catalyst to trust-building in a rapidly evolving emergency, particularly around the decisions, rules, expectations, and consequences for different industries, states, and species. Transparency in a complex, multi-species outbreak response should acknowledge both what is known (e.g., clear explanations of rationale behind decision-making) and unknown (e.g., recognizing policy drawbacks or scientific uncertainties). Examples include: why HPAI is considered an emerging disease in dairy cattle but a foreign animal disease in poultry; how H5N1 susceptibility and clinical illness differ among species and may require different control measures; that states vary in resources for managing outbreaks leading to different approaches; and why data is collected to inform and refine response measures.

While stakeholders may agree conceptually on transparency, it can be challenging to implement. Producers and workers may understand the importance of reporting illness amongst themselves or in their animals but still be reticent to do so out of uncertainty or fear of the consequences. Restricting visitor (including media) access to production units for biosecurity purposes may be misconstrued as signs of covert wrongdoing, prompting questions of how to implement “glass walls” that protect health and safety while still demonstrating openness. Open conversations, with early and iterative engagements throughout the outbreak, among producers and health authorities about what is known or remains uncertain on viral transmission routes, risk factors for infection, and potential control measures help reinforce trust between parties. Transparency and honesty in communication also promote understanding that science evolves and lend trust to possible changes in recommendations, which can increase the health authorities’ perceived benevolence and integrity, which are factors that affect a party’s trustworthiness (8).

## Incentives to support trust in interventions

Avian influenza and the associated control measures have significant health, social, and economic ramifications. Agricultural producers may experience loss of animals and products, costs for depopulation and quarantine, loss of customers and market share, and reputational damage. Farm workers may experience the economic impacts from lost wages, temporary or permanent job loss, and unpaid or reduced-pay leave time for educational sessions or health testing. These potential outcomes are perceived risks that may impact risk-taking behavior in the relationship between the producer and the government (8). As a result, these perceived risks can discourage engagement with infection control interventions and are exacerbated if producers and workers do not see benefits or trust the processes.

U.S. agriculture relies heavily on foreign workers: an estimated 51% of hired dairy workers were immigrants (18). In addition to potential language barriers which may constrain adherence with risk reduction interventions such as using personal protective equipment, workers may have concerns about engaging with government officials. This reticence may also hinder compliance with control measures such as travel restrictions if working at multiple locations. For farm workers, incentives from small snacks to cash rewards or gift certificates can encourage participation in educational sessions, testing programs, and illness reporting. Improved access to health care and paid sick leave can facilitate compliance with control measures among ill farm workers. For farm owners, benefit-sharing from research activities that help inform disease control in their operations can encourage cooperation with federal and state authorities. In addition, indemnity and compensation programs that appropriately cover losses for production, depopulation, and replacement; are equitably distributed; and are announced early in an outbreak are particularly important to support use of stringent disease control measures.

## Conclusion

Building trust for outbreak response must recognize that stakeholders have different experiences, resources, and roles that influence how they respond. Given that trust is context specific and is updated based on past outcomes (8), empowering stakeholders through iterative engagement, transparent communication, and incentives that lower their perceived risk for engaging with health authorities, will support trust building that will facilitate outbreak responses in the future. In the HPAI H5N1 outbreak in the U.S., there have been localized successes in proactive communication and cross-sector mobilization with industry partners by state health officials to build trust among the relevant stakeholders (19). However, these success stories could be replicated more broadly in the agricultural sector, and public health preparedness efforts could engage producers’ perspectives in simulation exercises to support operational learning and lay the foundation for building trust with these stakeholders for future outbreaks (20). These pillars for trust-building are foundational for instilling resilience into the U.S. food production system to protect the health and wellbeing of the nation’s livestock and people.

## Data Availability

Publicly available data and information were analyzed in this article. These materials can be found here: https://www.nationalacademies.org/units/HMD-BGH-18-P-16/event/44692.
